# Spatiotemporal distribution of essential elements through *Populus* leaf ontogeny

**DOI:** 10.1093/jxb/erw111

**Published:** 2016-03-16

**Authors:** Mónica R. Carvalho, Arthur Woll, Karl J. Niklas

**Affiliations:** ^1^School of Integrative Plant Sciences, Plant Biology Section, Cornell University, Ithaca, NY 14853, USA; ^2^Cornell High Energy Synchrotron Source, Cornell University, Ithaca, NY 14853, USA

**Keywords:** Calcium, essential elements, leaf development, leaf maturation, phloem loading, *Populus*, X-ray fluorescence spectroscopy.

## Abstract

We examined the spatiotemporal distribution and accumulation of calcium (Ca), potassium (K), and zinc (Zn) during the growth and maturation of grey poplar (*Populus tremula* × *alba*) leaves covering plastochrons 01 through 10. This period spans the sugar sink-to-source transition and requires coordinated changes of multiple core metabolic processes that likely involve alterations in essential and non-essential element distributions as tissues mature and effect a reversal in phloem flow direction. Whole-leaf elemental maps were obtained from dried specimens using micro X-ray fluorescence spectroscopy. Additional cross-sections of fresh leaves were scanned to check for tissue specificity in element accumulation. The anatomical distribution of Zn and K remains relatively consistent throughout leaf development; Ca accumulation varied across leaf developmental stages. The basipetal allocation of Ca to the leaf mesophyll matched spatially and temporally the sequence of phloem maturation, positive carbon balance, and sugar export from leaves. The accumulation of Ca likely reflects the maturation of xylem in minor veins and the enhancement of the transpiration stream. Our results independently confirm that xylem and phloem maturation are spatially and temporally coordinated with the onset of sugar export in leaves.

## Highlight

Calcium, potassium, and zinc have a preferential spatiotemporal distribution throughout leaf development, and compartmentalization of calcium in poplar leaves enhances the evapotranspiration stream and phloem maturation.

## Introduction

Essential elements such as calcium (Ca), potassium (K), and zinc (Zn) are required for adequate plant growth and reproduction (Hawkesford *et al.*, 2012). Along with other non-essential elements, these are taken up by roots from the soil and are subsequently moved into the shoot by long distance transport (White, 2011). Once within the shoot, their distribution and compartmentalization varies both spatially and temporally across organs and tissues, following metabolic requirements and uptake availability (Conn and Gilliham, 2010).

Element requirements and tolerance widely vary at the tissue, cellular, and subcellular levels. Whereas some elements exist within a broad concentration range, others become toxic if they accumulate beyond a critical level, indicating that metabolic controls must sense and regulate the targeted translocation of elements to maintain plant function. Transport proteins found in xylem and phloem tissues have been shown to mediate the movement of some essential elements into and out of the vascular system of several species (Yamaji and Ma, 2014; Zhai *et al.*, 2014). However, the specific mechanisms and timing behind the preferential distribution and accumulation of most essential elements between cell types in plants remain poorly known (Yamaji and Ma, 2014). The documentation of spatiotemporal patterns of essential element accumulation at the organ and tissue levels is necessary for gaining deeper insights into mineral distribution, sequestration, and compartmentalization of essential elements in plants.

Our knowledge of the distribution of elements at a macroscopic level has greatly increased in the past two decades with the application of numerous techniques that allow fast *in situ* detection and quantification (Wu and Becker, 2012; Zhao *et al.*, 2014). Interest in the accumulation of heavy metals has driven the application of less traditional methods such as energy dispersive X-ray microanalysis and proton-induced X-ray emission for detecting the distribution of macronutrients or heavy metals in hyper-accumulating plants at the cellular level. However, mapping the distribution of non-metalloid and/or lighter elements at low concentrations remains challenging and requires analytical methods that provide both high sensitivity and high spatial resolution. Synchrotron-based techniques using high-energy photon beams that allow for the detection of a wide array of elements have proved useful for visualizing element distribution at the tissue, cellular, and subcellular levels. Recently developed detector systems for synchrotron-based micro X-ray fluorescence (µ-XRF) spectroscopy have enhanced detection speed as well as the ability to spatially resolve elements in low concentrations (Kirkham *et al.*, 2010). This method has been successfully used to document *in situ* distributions of elements in commercially important crop plants, including *Triticum aestivum* (wheat; Regvar *et al.*, 2011), *Oryza sativa* (rice; Moore *et al.*, 2014), and *Citrus* × *paradise* (grapefruit; Tian *et al.*, 2014), and in the hyper-accumulator *Noccaea (Thlaspi) praecox* (Koren *et al.*, 2013).

Here, we make use of synchrotron-based µ-XRF to explore the temporal and spatial accumulation of essential elements in developing leaves of grey poplar (*Populus tremula* L. × *P. alba* L.). As in many other species, the leaf primordia of this species act as sinks that rely on carbohydrates and other nutrients unloaded from long distance phloem transport. As leaves grow and photosynthetic rates increase, sink strength decreases and leaves become net sources of photosynthates to newer developing leaves. This sink-to-source transition denotes the irreversible loss of import capacity and the initiation of net carbon export from leaves as they reach full maturity. This process requires a shift in transport direction and involves major changes in central metabolism, enzymatic machinery, symplastic connectivity, and anatomical modifications that are expected to correlate with element provenance, requirements, and allocation (Turgeon, 2006).

The distribution of essential elements in leaves is expected to match metabolic requirements throughout leaf ontogeny. The sink-to-source transition in developing leaves provides a system in which preferential element distribution can be followed in relation to the mechanical and metabolic changes associated with leaf growth. Because most developing tissues have low transpiration rates, young leaves are mostly unlinked from the transpiration stream and must rely on phloem transport for delivery of nutrients and mineral elements. As sink strength decreases, an increasing proportion of the inorganic nutrients entering the leaves come directly from the roots and through the xylem (Milthorpe and Moorby, 1969). Net concentrations of essential elements in leaves vary through development as cell division ceases and differentiation initiates. For example, phosphorous distribution and accumulation in developing leaves of *Cucumis sativus* has been well documented to reach a maximum concentration before the sink-to-source transition occurs and subsequently declines due to net export initiation (Hopkinson, 1964). Contrastingly, the targeted translocation of xylem-transported elements such as Ca from fully mature leaves is unlikely given that specific transport proteins mediating their export have not yet been found in phloem and that Ca specifically is known to have low phloem mobility. The accumulation patterns differ between mineral elements (Meiri *et al.*, 1992; Karley *et al.*, 2000; Karley and White, 2009) and are likely to reflect nutrient function, tissue specificity, and differences in transport pathways. However, the preferential distribution and temporal behaviour of most mineral elements in leaf tissues remains unknown.

Our goal in this study was to examine the spatiotemporal distribution and the relative abundance of mineral elements throughout leaf growth and maturation. We describe the distribution of Ca, K, and Zn in leaves of grey poplar, making reference to leaf growth, anatomical development, and the photosynthetic sink-to-source transition. The distribution of these three elements were emphasized because K is the most abundant cation in plants, Ca has low mobility in the phloem, and Zn is a well-studied trace-element model.

## Materials and methods

### Plant material and growing conditions

Grey poplar (*Populus tremula* × *alba*) was selected as a model species because it belongs to a species complex whose leaf development has been extensively studied and in which the use of leaf plastochrons provides precise ontogenetic control for understanding leaf development (Dickmann, 1971; Larson and Isebrands, 1971; Larson *et al.*, 1972; Isebrands and Larson, 1973; Dickmann and Gordon, 1975; Dickson and Larson, 1981). It was also selected because it employs passive rather than active phloem loading, which makes inferences about essential element transport more transparent (Zhang *et al.*, 2014).

Clones of grey poplar were propagated *in vitro* from stem cuttings rooted in Cornell mix growing medium (Boodley and Sheldrake, Jr., 1982) and grown in a growth chamber on 12h/12h day/night cycles under 300 µmol photons/m^2^ and day/night temperatures of 28°C/23°C. Once the plants reached ~25cm in height (~3 weeks), they were transferred to a cold frame and grown under natural ambient conditions at Cornell University facilities in Ithaca, NY, USA, between June and July 2014. Plants were watered daily and were supplemented weekly with a 15-15-15 complete nutrient solution.

Leaves were harvested from saplings >60cm in height after reaching a leaf plastrochron index (LPI; Larson and Isebrands, 1971) of at least 20. Leaves spanning LPI 01–10 were selected to compare element distribution between early developing and fully mature leaves, and to capture the leaf phloem sink-to-source transition. Leaves were placed in a plant press for 3 days until fully dried. A set of these leaves, spanning LPI 01–07 and a fully mature leaf at LPI 10 collected from a single plant was selected for µ-XRF analysis. A duplicate collection of leaves was freeze-dried for comparing sample preparation techniques. In this case, the harvested fresh leaves were snap frozen in dry ice and lyophilized for 5 days.

Anatomical details and transverse images of leaves were obtained from free-hand sections through fully mature, fresh leaves at LPI 10. Transverse sections were observed under an Olympus BX60 microscope and photographed using a Sony Progressive 3CCD. Leaf area was calculated from high-resolution photographs taken using a Canon T2i camera and 60mm macro lens (Canon, Japan), and images were processed with ImageJ software (Rasband, 2014).

Even though one of the advantages of µ-XRF is its ability to detect trace elements *in situ* using fresh samples, using dehydrated samples nevertheless increases the sensitivity for trace elements by increasing the signal to background ratio. We tried two leaf dehydration methods to minimize water content in the sample and avoid possible noise caused by dehydration in long scans (>8 hours) of large leaves. Lyophilization of early developing leaves yielded poor results, possibly related to the collapse of undifferentiated cells. Elements in freeze-dried leaves appeared to form irregular streaks throughout the leaf lamina (Supplementary Fig. S1), contrasting with oven-dried samples in which element distribution followed well-defined leaf veins. Because freeze-dried leaf tissues may have suffered damage, all of the following results are based on observations made from the oven-dried samples.

### Elemental X-ray fluorescence

Dried leaves and transverse sections from fresh leaves were mounted between two layers of Kapton® polyimide film for elemental µ-XRF spectroscopy at the Cornell High Energy Synchrotron Source (CHESS) facilities. Two beamlines were used upon availability. Whole leaves were scanned at the F3 bending-magnet beamline, equipped with a double crystal Si(111) monochromator (ΔE/E ~10^–4^) and a single-bounce monocapillary lens (capillary PeB605; Huang *et al.*, 2014) that focused the beam to a spot size of 20 µm diameter. Leaf transverse sections were scanned at the G3 undulator beamline, which employs synthetic W/B_4_C multilayer monochromators (ΔE/E ~10^–2^) for increased flux. A different single-bounce monocapillary was used in this setup (s/n), which focused the beam to a spot size of 10 µm diameter. Fluorescence spectra were obtained at 11.2 keV using a 384-element Maia detector (Kirkham *et al.*, 2010) placed perpendicular to the incident beam and situated 2mm from the sample. Samples were scanned horizontally at 20 µm step intervals and fluorescence was captured for 1, 5, or 10ms per step on whole leaf and transversal section samples, as needed. The captured XRF spectra were analysed using the dynamic analysis method to obtain elemental maps using the software GeoPIXE v7.1 (Ryan, 2000). The incident flux was calibrated using reference films of known mass. This flux, in turn, was used to calculate elemental mass from XRF peak areas using a fundamental-parameters approach (Ryan *et al.*, 2014).

Elemental abundance, a dimensionless number that provides a metric for quantifying the spatial distribution of each element in leaves differing in size and age, was calculated in the following way. The elemental masses for Ca, K, and Zn for each leaf were retrieved as ppm per scanned spot area. Estimated mass per scanned spot area was retrieved assuming that cellulose was the major organic component within each scanned area. This assumption is consistent with prior stoichiometric measurements of intact eudicot leaves, which show that organic carbon is the most abundant element in leaves. The elemental abundance per surface area scanned was calculated by dividing ppm of each element per surface area by estimated dry mass per surface area. This procedure normalized the abundance of each element with respect to the abundance of total dry mass. Changes in actual leaf dry mass as a function of leaf development were additionally determined by weighing dried leaves.

It is important to note that, as in most eudicot leaves, poplar leaves manifest significant anatomical heterogeneity (resulting from differences in the volume fractions of air, cell walls, and cytoplasm). This heterogeneity in tandem with the fact that leaves were dehydrated precluded attempts to measure elemental concentrations in conventional units such as micromoles.

## Results

**Fig. 1.**
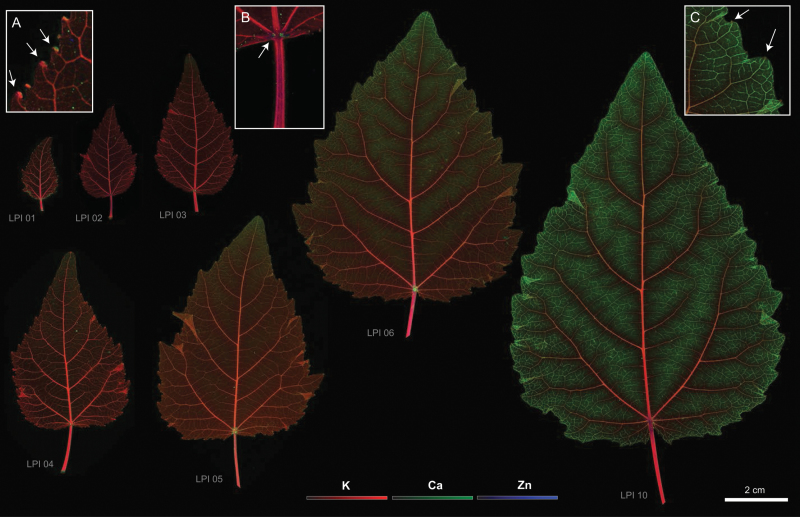
Composite µ-XRF maps of the abundance of Ca (green), K (red), and Zn (blue) (see colour gradients at the bottom of the figure) in developing leaves of grey poplar at LPI 01 (CHESS scan cycle/run 2014–2/102), LPI 02 (CHESS 2014–2/107), LPI 03 (CHESS 2014–2/120), LPI 04 (CHESS 2014–2/110), LPI 05 (CHESS 2014–3/1530), LPI 06 (CHESS 2014–3/1534), and LPI 10 (CHESS 2014–2/494). Fluorescence intensities of elements were normalized using reference standards, and provide a direct comparison of element abundance across representative leaves for each of the plastochrons shown (see centred scale at bottom for each of the three elements). Maximum pixel brightness corresponds to maximum abundance for each element. Note basipetal accumulation of Ca as leaves mature as shown by green colouration in leaves LPI 05, LPI 06, and LPI 10. Insert A highlights areas characterized by high abundance of Ca and Zn in teeth apices in LPI 01. Insert B highlights areas characterized by high abundance of Ca and Zn at the base of LPI 02 (see arrows). Insert C highlights the absence of co-localized Ca and Zn in teeth apices LPI 10 (see arrows).

**Fig. 2.**
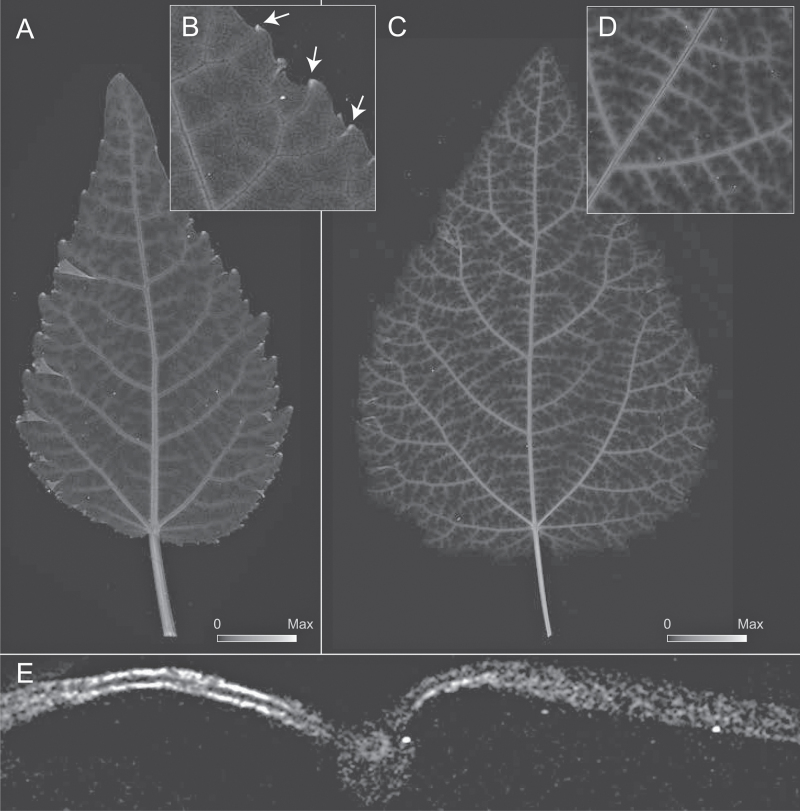
Elemental µ-XRF map for Zn abundance for developing leaves of grey poplar. Image intensity matches element abundance (see scales in A and C). **A** Early developing leaf at LPI 03. Maximum Zn = 1615 µg Zn/g dry mass. **B** Selected area from Fig. 2A highlighting the localization of Zn at leaf teeth apices (see arrows). **C** Fully mature leaf at LPI 10. Maximum Zn = 1305 µg Zn/g dry mass. **D** Detail of Zn localization around major veins at LPI 07. **E** Transversal section of a fully mature leaf at LPI 10 showing the accumulation of Zn in the upper and lower epidermis and in the peripheral vasculature of the midvein.

**Fig. 3.**
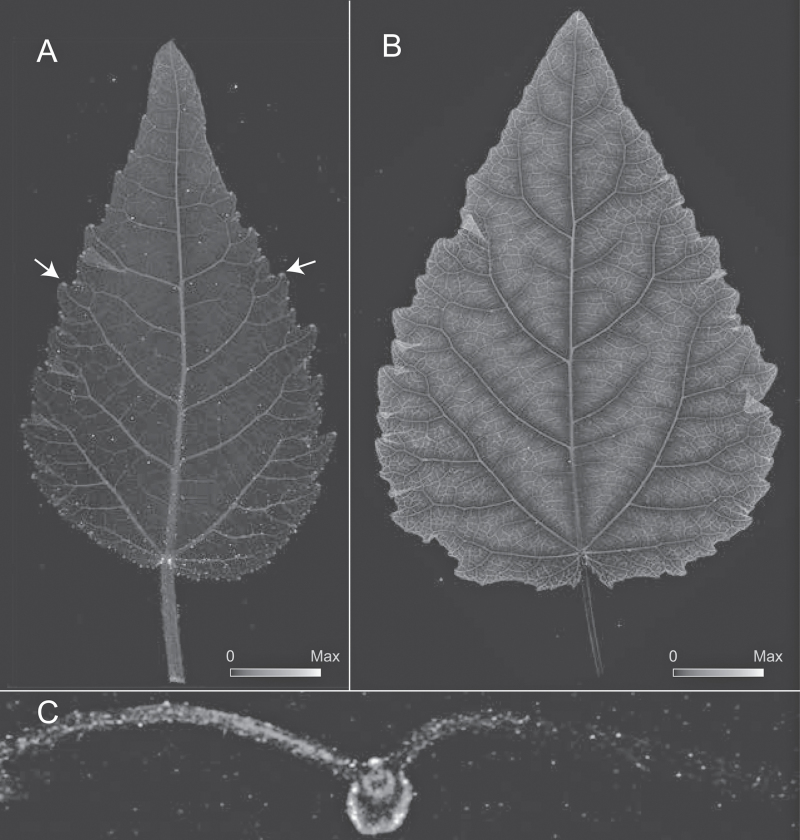
Elemental µ-XRF map for Ca for developing leaves of grey poplar. Image intensity matches element abundance (see scales in A and B). **A** Early developing leaf at LPI 03, showing Ca localization in teeth apices (see arrows) and major veins. Maximum Ca = 10625 µg Ca/g leaf dry mass. **B** Fully mature leaf at LPI 10 showing the localization of Ca in higher order veins and in the mesophyll as seen from above (see C for a transverse section). Maximum Ca = 10670 µg Ca/g leaf dry mass **C** Transversal section of fully mature leaf at LPI 10 showing Ca in mesophyll, the peripheral tissues in the midvein, and peripheral tissues of the midvein vascular strand.

Leaf elemental maps showed a distribution that varied both spatially across the leaf blade and temporally as gauged by elemental distributions from LPI 01 to LPI 10 (Fig. 1). We focus here on Ca, K, and Zn distributions because these elements manifested the most noticeable changes throughout leaf development (see Supplementary Fig. S2 for the elemental distributions of additional elements detected). The images shown in Fig. 1 reveal the distributions and abundances of all three of these elements and the ontogenetic changes in these variables of interest. The spatial distribution of each of the three elements during different stages of leaf development are discussed in detail below (and are shown in Figs. 2–3 and Fig. 5). The co-accumulation of two or more elements obscures the distribution of single elements. For example, the co-accumulation of K and Zn obscures the accumulation of each of these elements particularly in the apices of lamina teeth and the basal regions of juvenile leaves (Fig. 1A, B).

### Zinc distributions in leaf development

Elemental maps indicated the preferential distribution of Zn adjoining the leaf vasculature (Fig. 2A–E). In early developing leaves, the highest Zn abundance was observed at the apices of leaf teeth (Fig. 2B), in the bundle sheath surrounding the midvein and secondary veins (Fig. 2A–B), and in the upper and lower epidermis. Similar distribution patterns were observed for leaves between LPI 01 and 04, but the accumulation of Zn decreased in teeth apices with increasing LPI (compare Fig. 1A, B with Fig. 1C). Fully mature leaves (at LPI 10) no longer exhibited high Zn abundance in teeth apices (Fig. 2C), but showed a more pronounced Zn accumulation surrounding higher order veins and, once again, in the upper and lower epidermis (Fig. 2D, E). As leaves matured, Zn continued to accumulate in these regions, leaving a ghost-like image pattern in the midvein and higher order vascular strands owing to the greater abundance of Zn in these cells (Fig. 2C–E). Because transverse sections made from fresh leaves and immediately mounted for analysis yielded the same patterns (albeit with lower spatial resolution), it was unlikely that Zn accumulation around the vasculature was due to element displacement towards the veins during whole leaf dehydration.

### Calcium distributions in leaf development

**Fig. 4.**
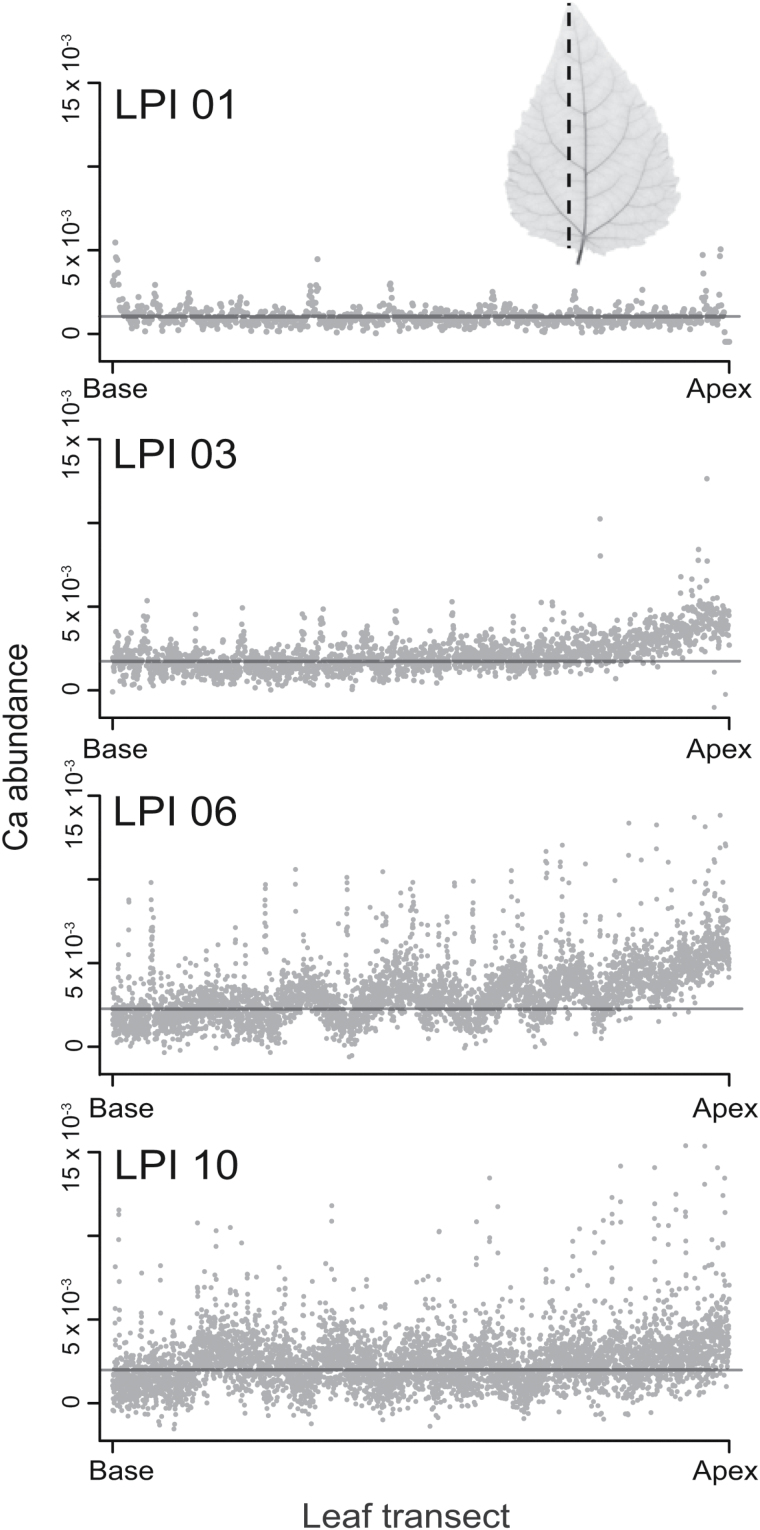
Variation in Ca abundance (i.e. g Ca/g dry mass) along longitudinal transects across grey poplar leaves in different stages of development (LPI 01, LPI 03, LPI 06, and LPI 10) (see dashed line in upper insert for transect orientation across each leaf). Solid lines in each graph indicate average leaf Ca abundance for each leaf. Ca abundance peaks in each graph correspond to the locations of major veins; peak maxima increase as leaves mature from LPI 01 to LPI 10. Ca accumulation occurs basipetally (from the apex to the leaf base) (see also Fig. 1).

Elemental maps indicated that Ca was highly concentrated in major veins as well as in teeth apices of early developing leaves (see arrows in Fig. 3A). Whereas the average abundance of Ca in major veins of the LPI 01 leaf was one-half that in teeth apices, the abundance of Ca within the leaf lamina was approximately one-half that of the midvein. The enhanced accumulation of Ca in major veins is observed as peaks in a wave-like pattern of Ca abundance along the leaf longitudinal transects shown in Fig. 4. A basipetal pattern of Ca accumulation is also indicated in Fig. 4 in the form of an increase in peak maxima towards the leaf apices of LPI 03 and LPI 06.

With increasing LPI, the marked difference in Ca abundance between teeth apices and leaf veins decreased, and the accumulation of Ca in the lamina became more noticeable (see Fig. 3B; see also Fig. 1). At LPI 03, the accumulation of Ca in the laminar tissues as well as in minor veins increased towards the apex (Fig. 3A), as illustrated by the wave-like pattern in the longitudinal Ca abundance transect shown in Fig. 4. Contrastingly, the abundance of Ca in leaf teeth apices decreased at LPI 03 (see Fig. 1). By LPI 06, more than half of the leaf lamina shows increased amounts of Ca (Fig. 4). The increasing frequency of peaks observed in the Ca abundance transect shown for LPI 06 in Fig. 4 indicates an enhanced accumulation of Ca in corresponding minor veins towards the leaf apex. At this developmental stage, the abundance of Ca in leaf teeth no longer differed from that of surrounding tissues (see leaf at LPI 06 in Fig. 1).

Fully mature leaves showed a different distribution pattern compared to that of early developing leaves (Fig. 3B). Whereas Ca accumulation was noticeable only in major veins of early developing leaves, the Ca abundance in veins of third, fourth, and even fifth order in fully mature leaves averaged twice that in primary and secondary veins (Fig. 3B). The difference in Ca abundance between the major veins and the lamina was not as marked. Specifically, Ca abundance in the lamina was ~20% lower than that in major veins, compared to a 50% difference observed in early developing leaves (see Fig. 3A, B). The maximum Ca abundance for all non-vascular leaf tissues was highest between secondary veins and lowest in proximity to primary and secondary veins. A leaf cross-section that included the primary veins indicated that Ca is localized in the bundle sheath and the external-most regions of the midvein (Fig. 3C). Outside these regions, Ca was distributed within the mesophyll.

### Potassium distributions in leaf development

**Fig. 5.**
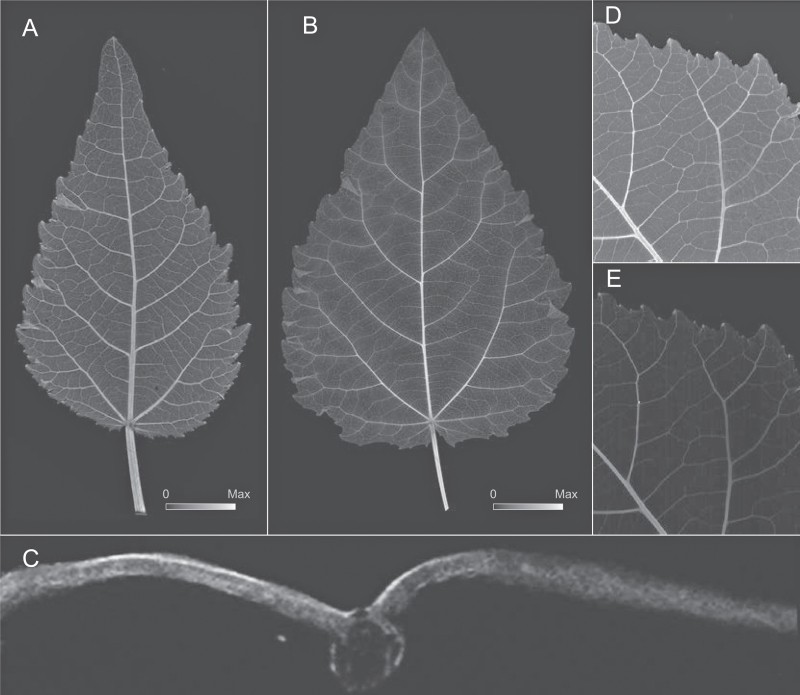
Elemental µ-XRF map for K for developing leaves of grey poplar. Image intensity corresponds to element abundance (see scales in A and B). **A** Early developing leaf at LPI 03. Maximum K = 57290 µg K/g leaf dry mass. **B** Fully mature leaf at LPI 10. Maximum K = 55900 µg K/g leaf dry mass. **C** Transverse section of fully mature leaf LPI 10 showing accumulation of K in the upper epidermis and mesophyll. **D** Detail of K accumulation in a developing leaf at LPI 04. **E** Cellulose distribution as shown in D based on predicted leaf thickness (using µ-XRF data and assuming cellulose as the primary source of dry mass absorption). In D and E, the abundance of K in veins appears high because the veins are denser than lamina tissues.

The distribution of K in leaves was far more homogeneous than that observed in the elemental maps of Zn or Ca, and was largely invariant across leaf development (Fig. 5A–C). K was more abundant in all vein orders than in the lamina, but its abundance decreased with increasing vein order (Fig. 5A, B). This pattern was observed in both early developing and fully mature leaves (Fig. 5A, B). The difference in K abundance between the lamina and veins was most pronounced in fully mature leaves, and was 10 times lower in the lamina than in the larger veins. We compared vein thickness to K abundance in veins and found that it did not fully match estimates of leaf thickness (compare Fig. 5D and Fig. 5E). The internal distribution of K indicated that this element was much more prevalent in the epidermis, mesophyll, and tissues surrounding veins than in either the xylem or phloem (Fig. 5C).

### Developmental changes

**Fig. 6.**
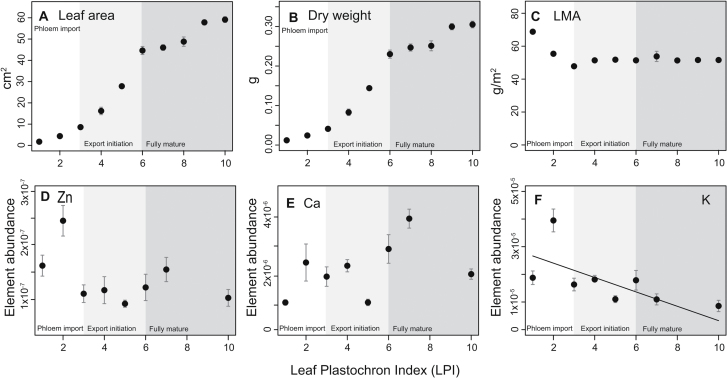
Leaf growth parameters and changes in element abundance (i.e. g element/g dry mass) in relation to leaf development and the transition from phloem importing to phloem exporting, as denoted by the shaded blocks (see notation at the bottom of each graph). **A** Leaf area. **B** Leaf dry weight. **C** Leaf mass per area (LMA). **D** Zn abundance. **E** Ca abundance. **F** K abundance (diagonal line denotes ordinary regression line for abundance vs. LPI).

Poplar leaf growth conforms to a sigmoidal curve with an early exponential increase in leaf mass and area that plateaus once leaves reach plastochron 10. Leaf mass per area decreased in early development and reached a relatively constant value by plastochron 3 (Fig. 6A–C).

The abundance of elements followed different patterns throughout leaf development. Whereas the total abundance of Ca, K, and Zn increased as leaves expanded to full maturity, the relative abundance of these elements varied (Fig. 6D–F). Zinc abundance decreased throughout leaf development, though this decrease was not statistically significantly (Fig. 6D). Ca abundance remained fairly constant throughout the leaf plastochrons examined in this study (Fig. 6E). In contrast, the average abundance of K in leaves decreased significantly with leaf growth (*r* ^2^ = 0.40, *P* = 0.05) (Fig. 6F).

## Discussion

Developing leaves undergo a number of structural and biochemical changes as they reach full maturity. Here, we document the preferential spatiotemporal distribution of Ca, K, and Zn throughout a developmental sequence at the whole organ level, and correlate the compartmentalization of Ca in leaves with phloem maturation. The conversion from sink to source marks a fundamental transition in leaf physiology that involves the coordinated decline of respiration and leaf growth rates, and an increase of carbon fixation rates needed to create a positive carbon balance and reverse the direction of phloem flow (Dickmann, 1971; Turgeon, 2006). These metabolic changes require the formation of functional stomata, substomatal chambers, and intercellular spaces that enable gas exchange, as well as the maturation of minor veins that mediate sugar export from the leaf (Turgeon, 1989, 2006), which also provides the driving force for transpiration, delivering water and solutes via the xylem.

Changes in leaf anatomy have been well characterized in species of poplar and have been linked to photosynthetic import and export capacities using the LPI system (Dickmann, 1971; Larson *et al.*, 1972; Isebrands and Larson, 1973; Dickson and Larson, 1981). Our study contributes to this database by reporting that the spatial and temporal distributions of Ca, K, and Zn in grey poplar changes in a manner that is consistent with major metabolic and developmental transitions in leaf ontogeny.

### Calcium in the leaf lamina

Poplar leaf development resembles that of many other eudicots in that growth is both structural and expansive (Pantin *et al.*, 2012). Before LPI 0, the basic anatomical organization of the leaf lamina is established and leaf growth is determined by cell division and structural growth. Cell expansion begins once the leaf reaches LPI 0, and cell separation is noticeable only in the spongy mesophyll by LPI 01. At LPI 03, intercellular spaces connected to functional stomata are found only at the leaf tip and spatially coincide with enhanced photosynthetic rates and the onset of sugar export (Dickmann, 1971; Isebrands and Larson, 1973). The development of intercellular spaces and sugar export are coordinated spatially and proceed basipetally until leaf maturation is attained at LPI 06 (Fig. 6).

Calcium accumulation in minor veins at the leaf tip (Fig. 1) is spatially and temporally consistent with the onset and progression of sugar export from the leaf. The accumulation of Ca in minor veins corresponds to minor vein maturation and the enhancement of the transpiration stream required for the onset of photosynthate export. It has long been thought that Ca is exclusively transported through the transpiration stream because transpiration rates are closely associated with leaf Ca content (Fricke *et al.*, 1995; Storey and Leigh, 2004; Kerton *et al.*, 2009; Gilliham *et al.*, 2011). However, Ca is required for juvenile organ development and phloem is the primary transport tissue in juvenile organs, such as leaf primordia. As intercellular spaces develop and become connected to functional stomata during leaf expansion, transpiration and carbon fixation rates increase locally and enhance Ca availability in areas where the leaf is transitioning from sink to source. This process also reflects the functional maturation of xylem and phloem in minor veins, even though the formation of minor veins precedes the initiation of cell separation. The vascular strands that form and free-ending veins are continuously connected at the leaf apex by LPI 0 (Isebrands and Larson, 1973). However, the structural and functional development of sieve elements in minor veins is synchronized with sugar export, or briefly predates the initiation of sugar export (Fellows and Geiger, 1974).

Ca is an essential plant macronutrient, a crucial regulator of plant growth, and is involved in key structural and signalling processes (Hirschi, 2004). In leaves, Ca moves apoplastically and accumulates with leaf age (Fig.4). Free Ca content, however, is under tight intracellular control because it is involved in numerous signalling processes triggered by changes in its concentration at the nanomolar scale (McAinsh and Pittman, 2008). Because Ca is not redistributed through the phloem (Karley and White, 2009), assimilation into the cell wall and sequestration into trichomes, idioblasts, and cell vacuoles for long-term storage is essential for leaf function (Karley *et al.*, 2000; Karley and White, 2009). The distribution of Ca in mature poplar leaves reported here is consistent with other studies reporting the preferential accumulation of Ca in the mesophyll of eudicots leaves (Fig. 3) (Storey and Leigh, 2004; Vogel-Mikuš *et al.*, 2008; Kerton *et al.*, 2009; Conn *et al.*, 2011; Gilliham *et al.*, 2011). The Ca content in minor veins reported here is consistent with the accumulation of abundant calcium oxalate crystals, which are typically found in bundle sheath cells of poplar leaves.

### Leaf teeth apices, and calcium and zinc in early development

Noticeable amounts of Ca and Zn were associated with the hydathodes in major vein terminations of early developing leaves (Figs 1–3). As leaves grow, the high abundance of these elements decreases within hydathodes and matches those seen in major veins after LPI 04. This pattern is consistent with the increases in gas exchange and carbon fixation rates towards the margin of toothed leaves during early development (Baker-Brosh and Peet, 1997; Royer and Wilf, 2006). Specifically, highly transpiring leaf margins will experience increased water flux through major veins during early development, which will result in the accumulation of elements. However, this expectation does not fully explain the abundance of elements specifically in hydathodes or the decrease in elemental abundances attending leaf growth. An alternative scenario is that the accumulation of Ca and Zn is a by-product of guttation and the exudation of water out of the leaf, which results in the accumulation of elements near the leaf margin. A third possibility may involve an active accumulation of elements in hydathodes that enhances the osmotic potential at secondary vein terminations. Active transport of solutes from the transpiration stream and into phloem parenchyma and epithem cells of leaf teeth has been shown to occur in other species of poplar (Vogelmann *et al.*, 1985; Wilson *et al.*, 1988), and is thought to contribute to maintaining water flow velocities in xylem vessels (Wilson *et al.*, 1991). However, the mechanisms describing the targeted movement of ions into hydathodes have yet to be described. Further work is required to resolve which among these possibilities, if any, is correct.

### Potassium abundance and leaf age

K is the second most abundant cation in the cytosol and is functionally related to a broad range of core cellular processes, including pH maintenance, enzyme activation, cation/anion balance, and osmoregulation (Hawkesford *et al*., 2012). Our data show that K is preferentially located in the mesophyll of fully mature leaves, and that the average abundance of K decreases with leaf age (Fig. 6). K is typically prevalent in high concentrations in dividing tissues, where it is closely involved in the synthesis of structural proteins and in regulating turgor-driven expansion processes (Szczerba *et al.*, 2009). Long distance movement of K occurs through xylem and phloem. Given that early developing tissues have overall low transpiration rates, it is likely that most of the K present in young leaves passes through the phloem and is distributed throughout the leaf symplastically. As leaves grow and their sink capacity declines, import rates decrease, which can in part explain decreasing K abundances with leaf growth.

Most elements become more readily available in leaves as they mature and transpiration rates increase. Yet, most are typically remobilized to avoid hyper-accumulation, in response to metabolic demands in sink tissues, and during organ senescence. Plants growing under K-deprived conditions typically exhibit relocation of K from old to developing tissues. K is closely linked to sugar export; it contributes to the osmotic potential in phloem sap and is directly involved in sugar loading processes (Komor, 2000; Lalonde *et al.*, 2003). The expression of K^+^ channels in minor vein phloem is controlled by photosynthate supply (Deeken *et al.*, 2000) and may be involved in increasing net export rates for K as leaves grow and contribute to the net decline in abundance observed in grey poplar.

## Supplementary data

Supplementary data are available at *JXB* online.

Fig. S1. Elemental map of K obtained from a leaf at LPI 01 that was freeze-dried before µ-XRF scanning.

Fig. S2. Elemental µ-XRF map of additional elements (S, Cl, Cu, Mn, Fe) obtained from a fully mature leaf at LPI 10 of grey poplar.

Supplementary Data
