# Supplementary material for: Spatiotemporal distribution of essential elements through Populus leaf ontogeny
Source: J Exp Bot. 2016 Mar 16;67(9):2777–86. doi: 10.1093/jxb/erw111 (PMC4861023; doi:10.1093/jxb/erw111)
Supplement: Supplementary Data [file supp_67_9_2777__index.html]

Spatiotemporal distribution of essential elements through Populus leaf ontogeny — Spatiotemporal distribution of essential elements through Populus leaf ontogeny — Supplementary Data 

# Spatiotemporal distribution of essential elements through *Populus* leaf ontogeny

## Supplementary Data

Data files

- supplementary\_figures\_S1\_S2.pdf - Supplementary Data
