# Supplementary figures and images for: Spatiotemporal distribution of essential elements through Populus leaf ontogeny
Source: J Exp Bot. 2016 Mar 16;67(9):2777–86. doi: 10.1093/jxb/erw111 (PMC4861023; doi:10.1093/jxb/erw111)

**Figure S1**

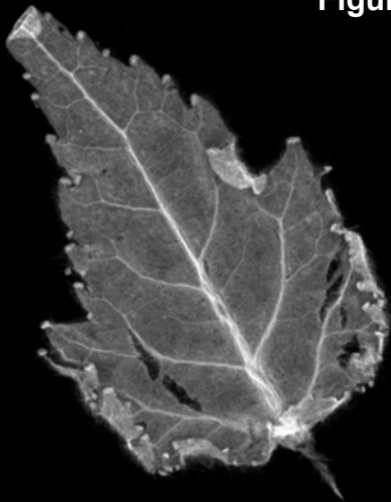

2 cm

**Figure S2**

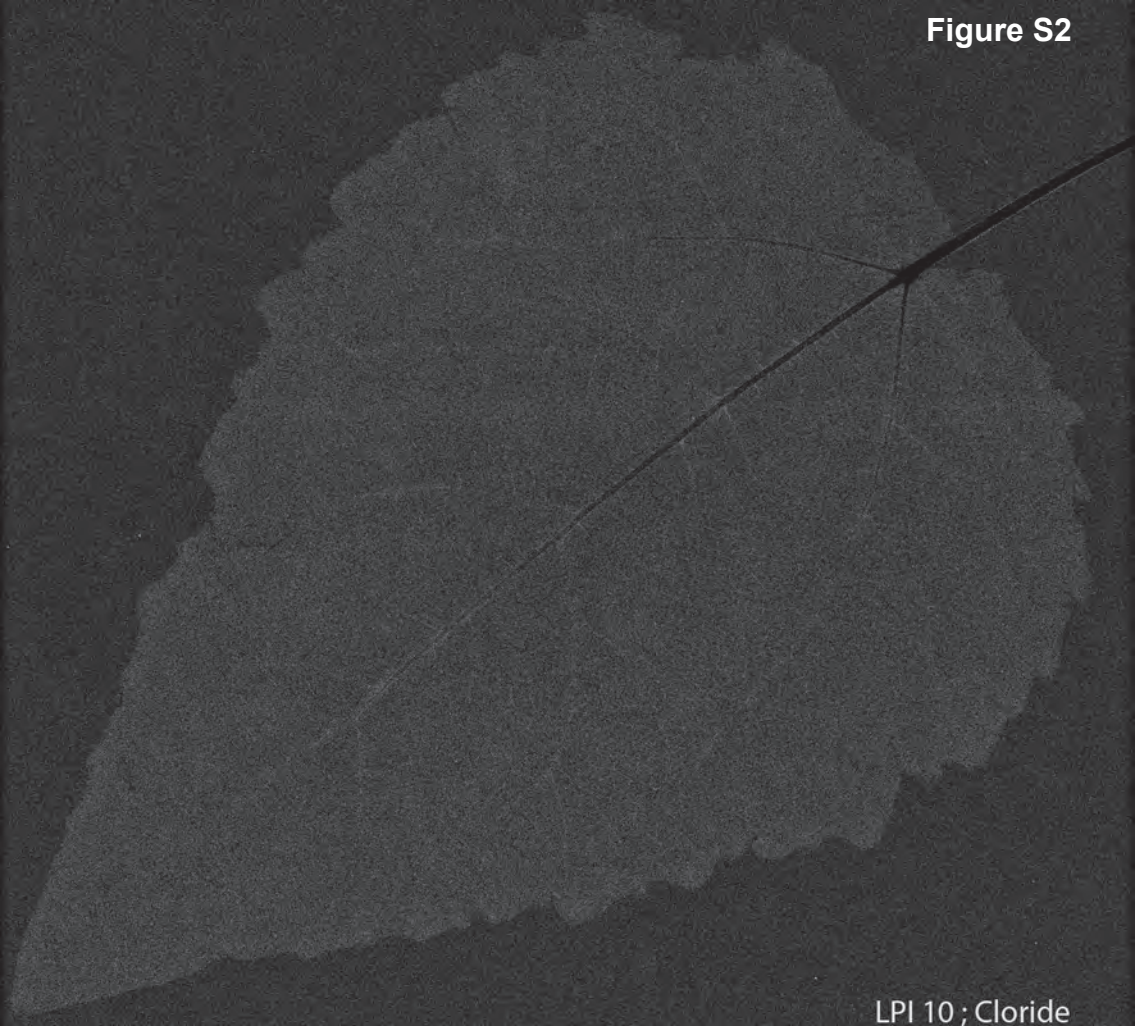

LPI 10 ; Cloride

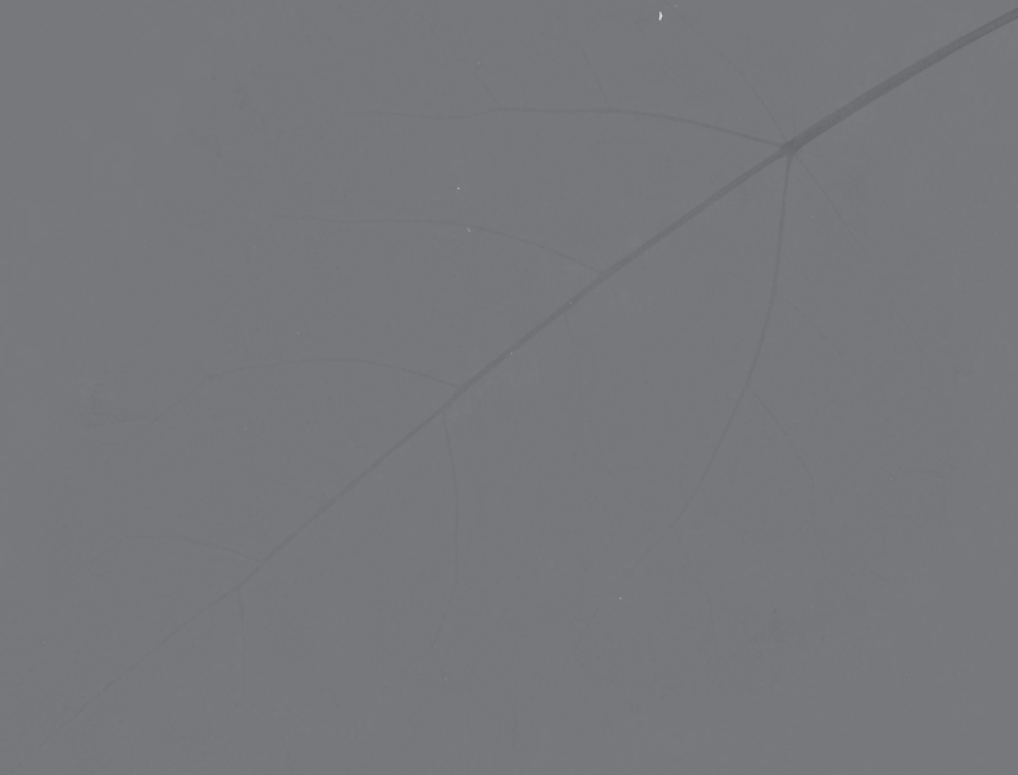

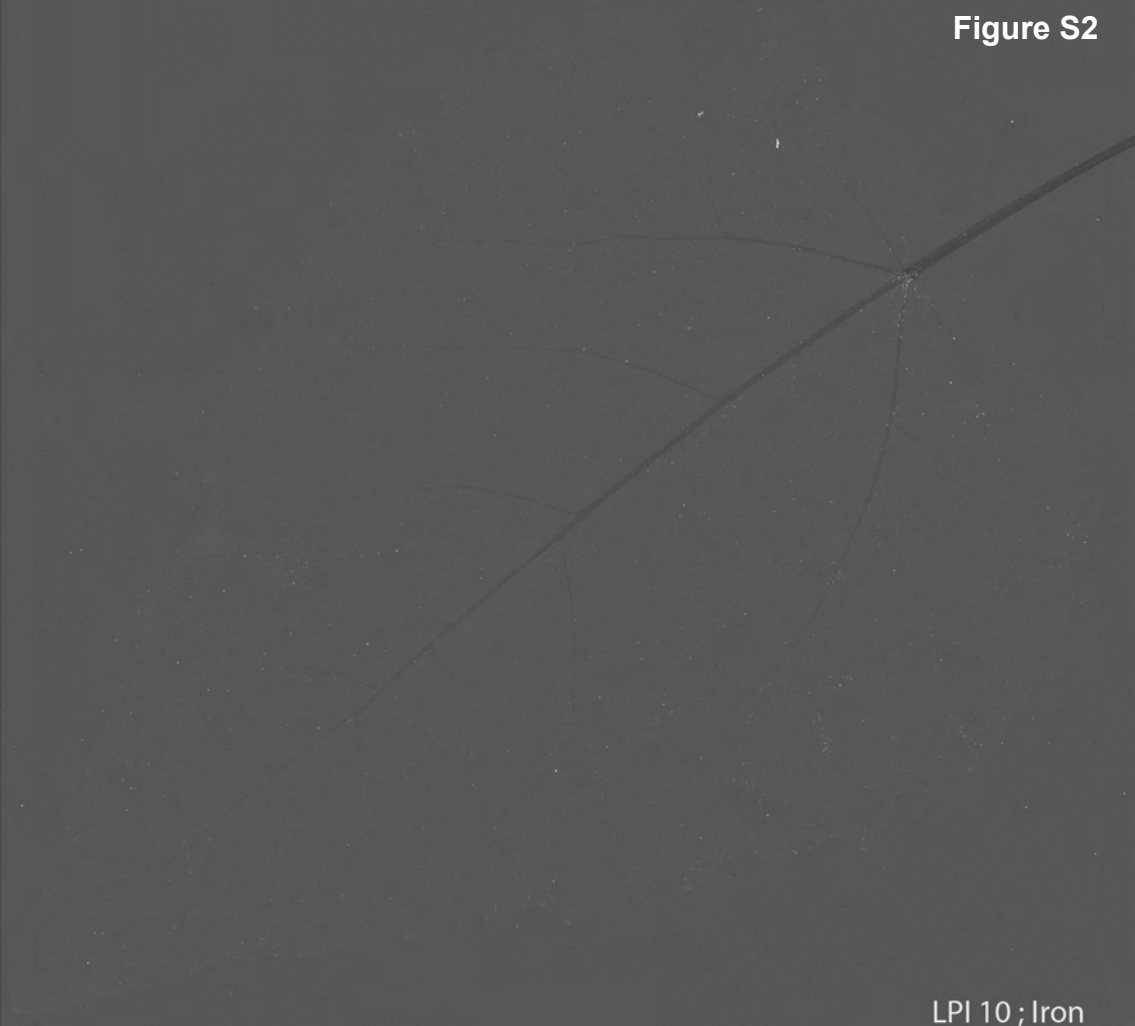

Figure S2

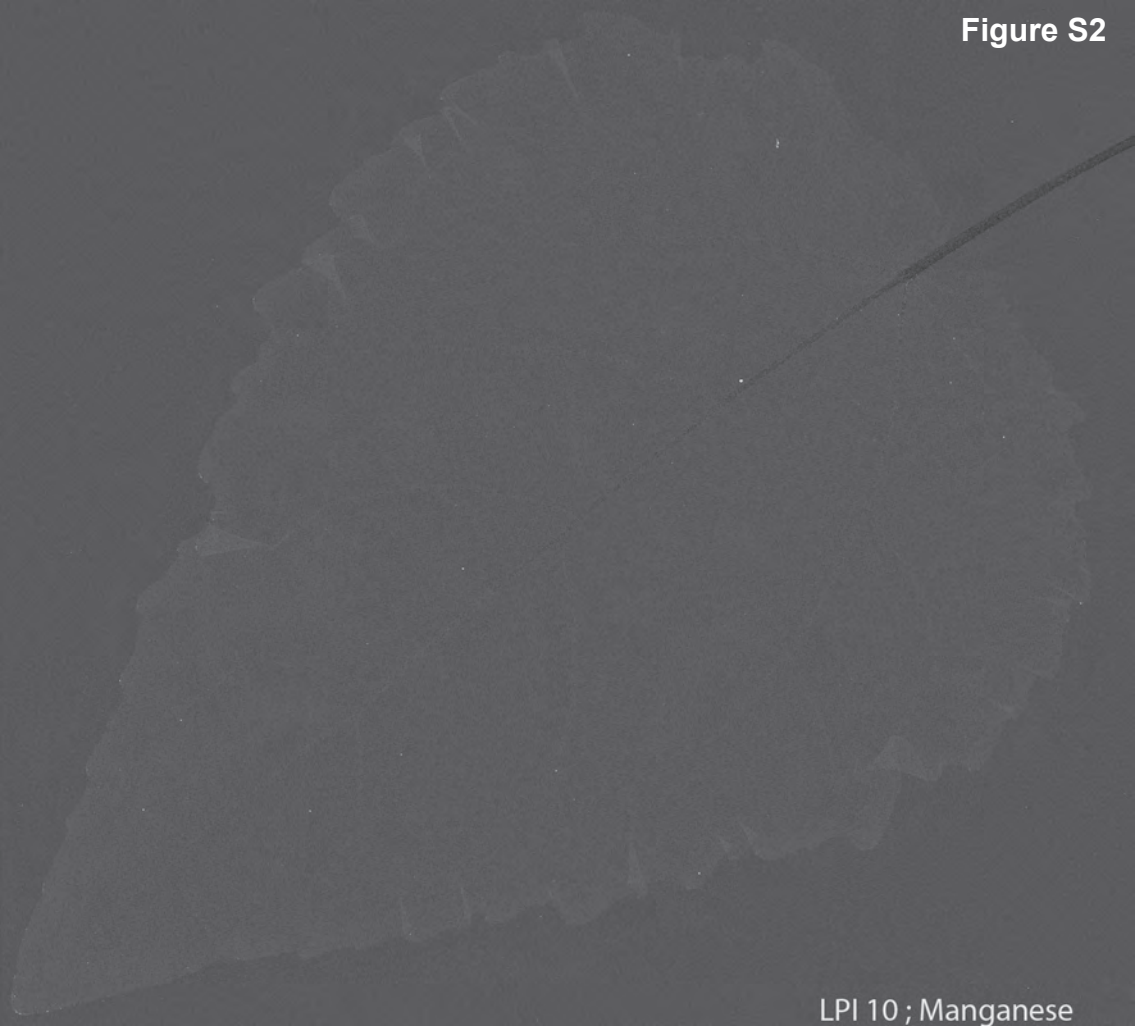

LPI 10 ; Manganese

Figure S2

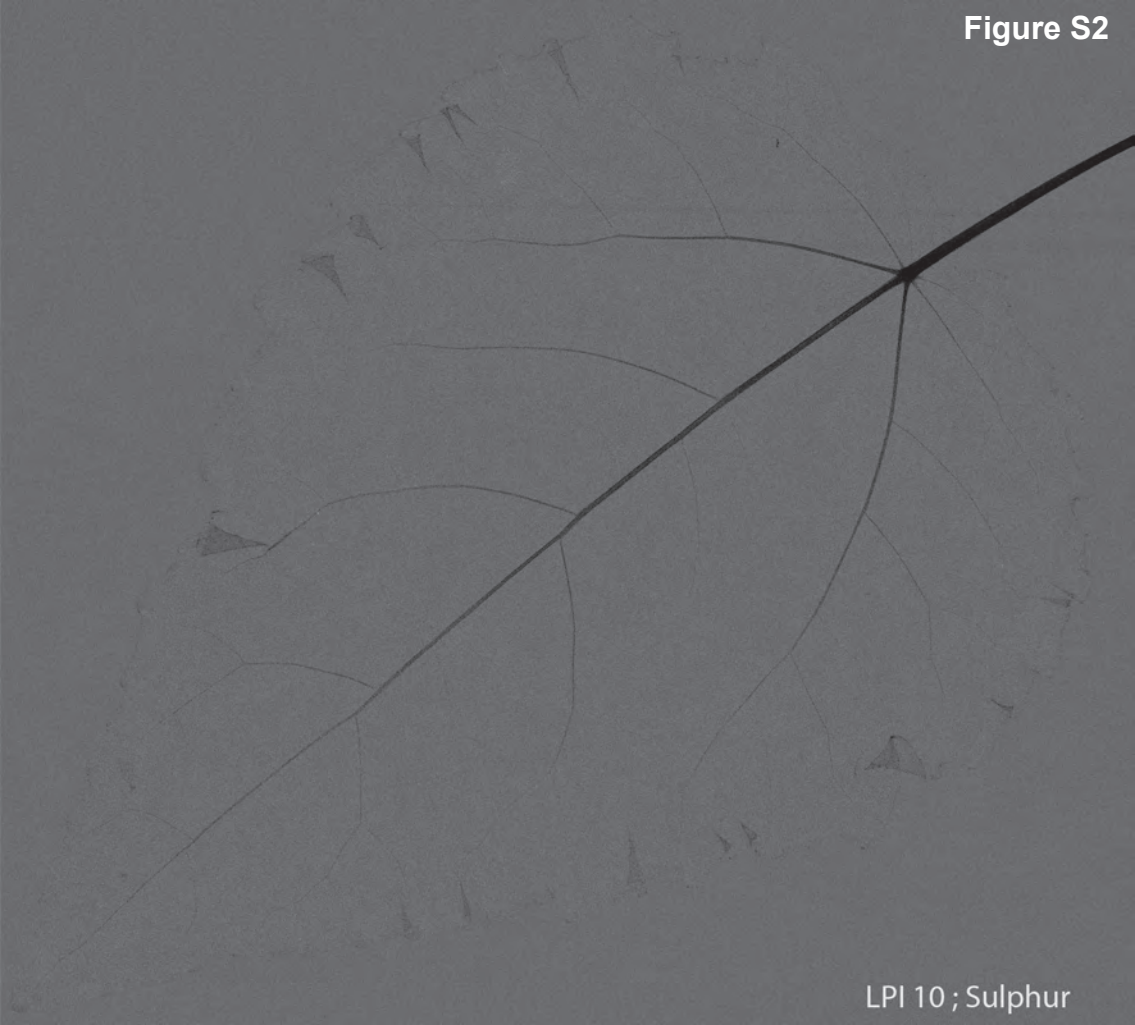

LPI 10 ; Sulphur

Supplement: Supplementary Data [file supp_erw111_supplementary_figures_S1_S2.pdf]
